# Phenotypic and Transcriptomic Response of Auxotrophic *Mycobacterium avium* Subsp. *paratuberculosis leuD* Mutant under Environmental Stress

**DOI:** 10.1371/journal.pone.0037884

**Published:** 2012-06-04

**Authors:** Jenn-Wei Chen, Joy Scaria, Yung-Fu Chang

**Affiliations:** Department of Population Medicine & Diagnostic Sciences, College of Veterinary Medicine, Cornell University, Ithaca, New York, United States of America; Naval Research Laboratory, United States of America

## Abstract

*Mycobacterium avium* subsp. *paratuberculosis* (MAP) is the causative agent of severe gastroenteritis in cattle. To gain a better understanding of MAP virulence, we investigated the role of *leuD* gene in MAP metabolism and stress response. For this, we have constructed an auxotrophic strain of MAP by deleting the *leuD* gene using allelic exchange. The wildtype and mutant strains were then compared for metabolic phenotypic changes using Biolog phenotype microarrays. The responses of both strains to physiologically relevant stress conditions were assessed using DNA microarrays. Transcriptomic data was then analyzed in the context of cellular metabolic pathways and gene networks. Our results showed that deletion of *leuD* gene has a global effect on both MAP phenotypic and transcriptome response. At the metabolic level, the mutant strain lost the ability to utilize most of the carbon, nitrogen, sulphur, phosphorus and nutrient supplements as energy source. At the transcriptome level, more than 100 genes were differentially expressed in each of the stress condition tested. Systems level network analysis revealed that the differentially expressed genes were distributed throughout the gene network, thus explaining the global impact of *leuD* deletion in metabolic phenotype. Further, we find that *leuD* deletion impacted metabolic pathways associated with fatty acids. We verified this by experimentally estimating the total fatty acid content of both mutant and wildtype. The mutant strain had 30% less fatty acid content when compared to wildtype, thus supporting the results from transcriptional and computational analyses. Our results therefore reveal the intricate connection between the metabolism and virulence in MAP.

## Introduction

*Mycobacterium avium* subsp. *paratuberculosis* (MAP) is a multispecies pathogen and is the etiological agent of a severe gastroenteritis in ruminants, known as Johne's disease [1]. The clinical symptoms of MAP infection in cattle include diarrhea, weight loss, decreased milk production, and mortality. Clinically or subclinically infected animals shed MAP in feces and/or milk enabling easy dissemination to other animals [1], [2]. MAP is also a concern in human health as it has been suspected as a possible cause of Crohn's disease in humans [3], [4].

Considering its obvious importance in animal industry and possible importance in human health, different strategies including use of DNA and recombinant antigen vaccines have been used in attempts to reduce the economic loss against MAP infection [5], [6], [7], [8]. Production of live attenuated strains of MAP with vaccine potential using transposon mutagenesis and allelic exchange has also been attempted [9], [10], [11]. Majority of these studies have utilized the advances in genetics research in related *Mycobacteria*, particularly *Mycobacterium tuberculosis*.

Several virulence and antigenic genes have been characterized in *M. tuberculosis*. The *leuD* gene which encodes isopropyl malate isomerase, an essential enzyme for leucine biosynthesis, is an example. In mice, the *leuD* mutant of *M. tuberculosis* has been demonstrated to be protective against challenge with virulent *M. tuberculosis* [12]. Similarly the leucine auxotroph of *M. bovis*, created by deletion of *leuD* using allelic exchange, was found to be protective in cattle against *M. bovis* challenge [13]. *leuD* knock-outs have been also used as auxotrophic selectable markers in *M. avium* and *M. smegmatis* [14], [15]. In another study focused on *M. bovis* BCG strain, *leuD* was found to be involved in an oxidative stress response [16]. *leuD* has been identified as part of PhoPR system in *M. tuberculosis* [17] and as a component of *codY* regulon in *Listeria monocytogenes* [18]. Recently we created a *leuD* knockout (MAPΔ*leuD*) strain using allelic exchange method [19]. We also demonstrated that MAPΔ*leuD* produced partial protection against MAP challenge [19]. Although these studies have clearly established the potential of knockout strain of *leuD* as a vaccine candidate, the role of *leuD* in virulence associated functions is largely unexplored. Since *leuD* is part of central intermediary metabolism, it will be particularly useful to understand how these pathways are involved in virulence associated functions. In this study, in order to elucidate the mechanism of *leuD* attenuation in MAP, the mutant and wildtype were characterized using phenotype array (PM) and the response of MAPΔ*leuD* was assessed by transcriptional profiling under different physiologically relevant stress conditions. We find that *leuD* metabolism is intricately connected with virulence associated genes and also fatty acid biosynthesis. Although we have detailed knowledge on molecular function of microbial virulence factors, our understanding of how nutrition of bacteria during infection controls these virulence genes is at its beginning. Therefore, the results of this study adds significantly to our understanding of how leuD biosynthetic pathway is connected to virulence functions.

## Materials and Methods

### Bacterial strains

As the genome of *M. paratuberculosis* strain K10 has been sequenced, we used this strain to develop a mutant of *leuD* gene by allelic exchange, using methods described previously [19]. MAP Wild type (MAP-WT) and MAPΔ*leuD* generated was grown in 7H9 medium supplemented with 10% oleic acid–albumin–dextrose–catalase(OADC) (Becton Dickinson Co., Sparks, MD) and 1.25 mg/L mycobactin J (Allied Monitor, Inc., Fayette, MO).

### Phenotype microarrays

Phenotype microarray experiments were performed following standard Biolog Inc. (http://www.biolog.com/) protocols [20], [21]. Briefly, MAP strains were grown on Middlebrook 7H10 agar at 30°C with supplementation of casitone and were resuspended in Biolog inoculating fluid so as to have 81% transmittance. Aliquots (100 µl) of this suspension were used to inoculate Phenotype microarray panels. Full metabolic profiles covering 760 tests were performed. Metabolite utilization at 37°C was measured every six hours up to 138 hours as color changes of redox dye G using an ELISA reader at 630 nm. Tests were performed in duplicate, and the mean signal intensity was calculated for each replicate. Each test well was then compared against negative control well of respective PM plate using ANOVA. Signal intensity increase of 25% or more from negative control at 5% significance level was considered positive phenotype.

### Stress experiments

Bacteria were grown to a cell density of 0.7 at OD_600_ in 7H9 broth with supplementation of casitone (7H9-casitone) and were subjected to the following three stressors for 3 hours (i) shift from 30°C to 42°C, (ii) shift from pH 7.0 to pH 5.5 and 9.0 and (iii) anaerobic incubation. Growth in medium without casitone was used as the fourth stress condition (minimal medium). Since *leuD* mutant could not grow without the supplementation of casitone in 7H9 broth, it was grown in 7H9-casitone broth as described above and was shifted to 7H9 without addition of casitone and was incubated for 2 days. Three biological replicates were conducted for each stress condition. At the end of stress experiments, twice the volume of RNA protect reagent was added to bacterial cultures; these were incubated at room temperature for 5 minutes. Ten milliliters of cells were then pelleted by centrifugation at 13,000 g for 5 minutes and were stored in −80°C.

### RNA extraction, cDNA synthesis, template labeling, array hybridization and data analysis

Cell pellets were transferred to a 2 ml sterile screw-cap microcentrifuge tube. 1 ml of TRIzol reagent (Invitrogen, Life Technologies) and 0.6 ml of sterile RNase-free 0.1 mm zirconia beads were added to each tube. Cells were homogenized and lysed by bead beating four times in a Mini Bead-Beater (BioSpec Products, Inc., Bartlesville, OK) for 30 seconds with a gap of 30 seconds. Cell lysate was transferred to a 1.5 ml sterile RNase-free microcentrifuge tube. RNA from the lysate was cleaned up using RNeasy MinElute Cleanup Kit (Qiagen, Valencia, CA). Before elution of RNA from the MinElute columns, following manufacturers protocols, possible contaminating DNA was removed using Qiagen RNase-Free DNase Set. Integrity of isolated RNA was estimated using Agilent Bioanalyzer 2100. Only those samples with an RNA Integrity Number (RIN)>8 was used for cDNA synthesis. 3.0 µg of RNA from each sample were reverse-transcribed using SuperScript III reverse transcriptase with random hexamers (Invitrogen) according to the manufacturer's instructions. The corresponding cDNA was labeled with 25 U of Klenow fragment (New England Biolabs, Ipswich, MA), 7 µg of exo-resistant random primers (Fermentas, Glen Burnie, MD), dNTP mix (0.12 mM each dATP, dTTP, and dGTP and 0.03 mM dCTP), and 0.1 mM of Cy3/Cy5-dCTP (Amersham Biosciences, Piscataway, NJ) at 37°C for 12 hours. Labeling reaction was cleaned up using a QIAquick PCR Purification Kit (Qiagen). A custom expression array for MAP K10 genome was designed using Agilent E-array algorithm (https://earray.chem.agilent.com/earray/). This array consisted of 60mers representing each CDS in MAP K10 genome [Gene Expression Omnibus (GEO) platform ID# GPL15269]. Following standard Agilent protocols, labeled cDNA was hybridized against this custom array. Cy3 labeled cDNA from wildtype strain served as control channel and Cy5 labeled *leuD* mutant cDNA from the corresponding stress condition served as the test channel. All three biological replicates were hybridized in duplicates. Arrays were then scanned using Agilent G2565BA microarray scanner and data was extracted using Agilent's feature extraction image analysis software v 10.2. The complete set of microarray files has been deposited in GEO (accession # GSE36053). Resulting data files were then loaded to GeneSpring GX v.11 (Agilent Technologies, CA). To allow interpretation of the data, values below 0.01 were set to 0.01. The log2 ratio of the intensity of signal channel to that of control channel was calculated and was then normalized to each slide's median intensity ratio. The default interpretation mode was set to ‘log of ratio’. Each time point was then tested separately for statistical differences using ANOVA at 5% confidence level, in conjunction with Benjamini-Hochberg multiple testing correction. From this list, those genes changing more than 1.5 fold and 2.0 fold were selected as differentially expressed genes (DEGs).

### Quantitative real-time PCR (qRT-PCR)

1.0 µg of the total RNA was converted to cDNA by using SuperScript III reverse transcriptase (Invitrogen) with random hexamers, following the manufacturer's instructions. The real-time reaction mixture included 5 µl of cDNA template, 200 nM of each of both forward and reverse primers, and 1× Power SYBR Green master mix (Applied Biosystems, Foster, CA). Primers used in this study are listed in the Table S1. RT-PCR was performed in 96-well optical plates using the ABI 7500 Sequence Detection System instrument and software (Applied Biosystems). The thermal cycling consisted of an initial denaturing step at 95°C for 10 min followed by 40 cycles consisting of 95°C for 15 s and 60°C for 1 min. For C_t_ determination, three parallels were assayed for each gene *and* MAP_4078 gene was used as the internal reference gene. qRT-PCR was performed in triplicate to evaluate reproducibility of data.

### Computational analysis

The interaction data for MAP was extracted from STRING database [22]. Since online version of STRING database supports only a maximum of 1,000 nodes, complete interaction data from STRING v 9.0 was downloaded as a full database dump. MAP specific interaction data was then extracted using custom Linux shell scripts. The interaction data in STRING database includes both experimental as well as predicted interaction information [22]. Interactions in STRING are provided with a confidence score. The interactions with a confidence score less than 700 were removed and this interaction network was then imported into Cytoscape [23] for visualization and overlaying of microarray expression data. Effect of *leuD* deletion in MAP pathways was analyzed using Pathway tools software [24], [25]. An Omics viewer [25] was used to visualize the differentially expressed genes on MAP pathways. Enrichment analysis implemented in Pathway tools was used to indentify statistically significant pathways associated with each stress condition. The mycolic acid biosynthesis pathway in MAP was deduced using *M. tuberculosis* pathway as reference. Takayama *et al.* has defined the *M. tuberculosis* genes associated with mycolate biosynthesis pathway [26]. This gene list was used as input for ortholog mapping tool in Integrated Microbial Genomes [27] to find the MAP orthologs. Result of this mapping is given in Table S2. For analyzing the effect of *leuD* deletion on the virulence, MAP virulence associated gene definitions were downloaded from the Virulence factor database (VFDB) [28] and were combined with microarray expression data.

### Analysis of fatty acids and fatty acid methyl esters (FAME)

Three 100-ml biological replicates of MAP-WT and MAPΔ*leuD* were grown to mid-exponential-phase in 7H9 medium. MAPΔ*leuD* was then shifted to 7H9 medium without casitone and incubated for 12 hours. MAP-WT was incubated in fresh 7H9 medium for the same duration. The respective replicates of both MAP-WT and MAPΔ*leuD* were combined, and the frozen pellets were submitted for FAME analysis at Microbial ID, Newark, DE (http://www.microbialid.com/). The FAME data was analyzed using Sherlock® V 6.1.

## Results

### Growth characteristics of MAPΔleuD

**Figure 1:**
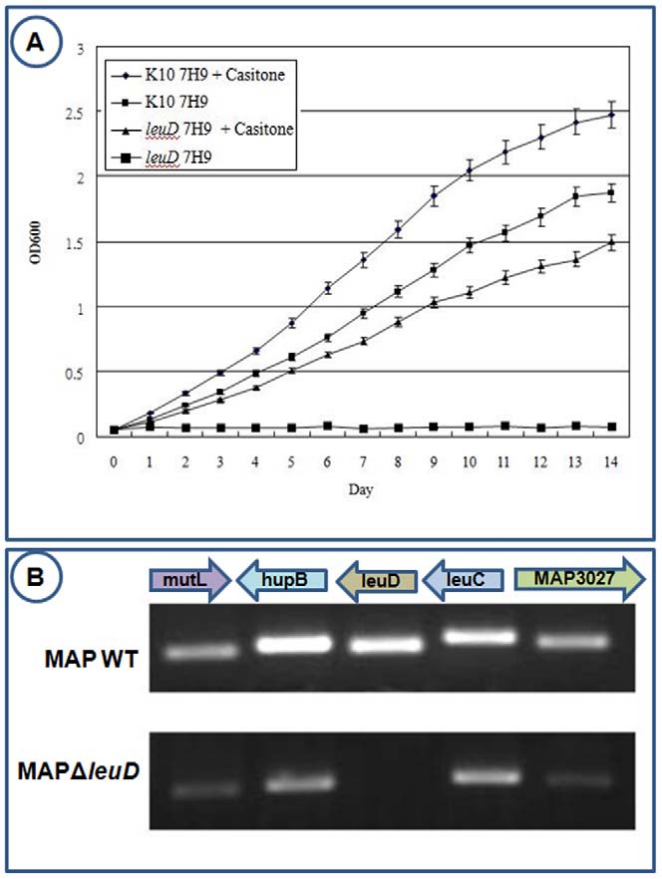
Growth characteristics of MAP-WT and *leuD* mutant. 1A. Both MAP-WT and *leuD* mutant were grown in 7H9 medium and 7H9 medium supplemented with casitone as source of leucine. Growth was measured as increase in turbidity of culture medium at OD600. 1B. qRT-PCR measurement of transcriptional levels of genes upstream and downstream of *leuD*. qRT-PCR products for each gene was loaded into a 1.2% agarose gel and was visualized using a gel documentation system.

Previously, we reported the development of *MAPΔleuD* strain and its potential as a vaccine candidate [19]. *MAPΔleuD* is an auxotroph of *leuD* which requires supplementation of 7H9 with Casitone in order to culture in that medium. Here, casitone was used as a leucine source since our strain had an impaired leucine biosynthetic pathway and cannot grow in un-supplemented 7H9 (Fig. 1A). With the addition of casitone, growth was restored albeit to a slightly less degree than the wild type strain (Fig. 1A). To confirm that the impairment of the growth was only due to the disruption of *leuD* but not due to the so called “polar effect” on the genes upstream or downstream, we compared the mutant and wildtype strains using qRT-PCR on *leuD* locus and adjacent genes. The results show that while the expression of *leuD* is completely abolished in MAPΔ*leuD*, expression of all other genes upstream and downstream are unaffected (Fig. 1B).

### Deletion of leuD leads to an impaired metabolic phenotype

**Figure 2:**
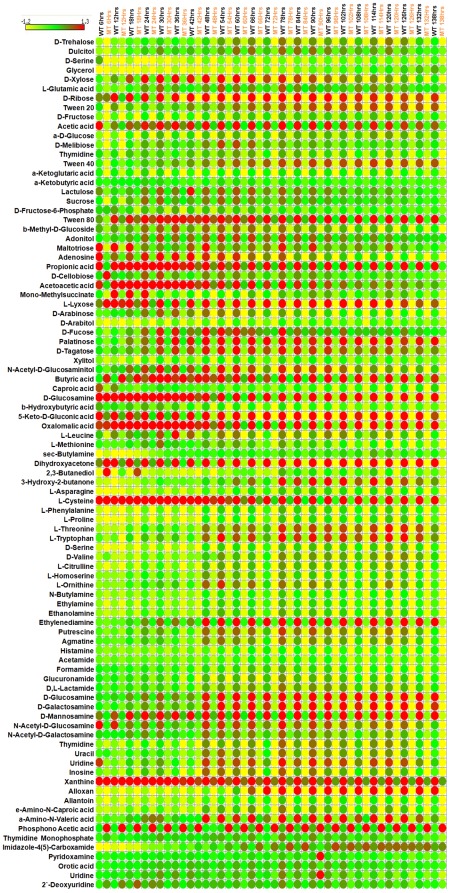
Phenotype array results for carbon, nitrogen, phosphorus, sulphur and nutrient supplements. Normalized values for positive wells in PMs 1–5 are presented. Time course measurement data for up to 138 hours is shown. Each row represents a compound and each column represents MAP WT or *leuD* mutant at a particular time point. Expression range is indicated at top left side of the figure.

**Figure 3:**
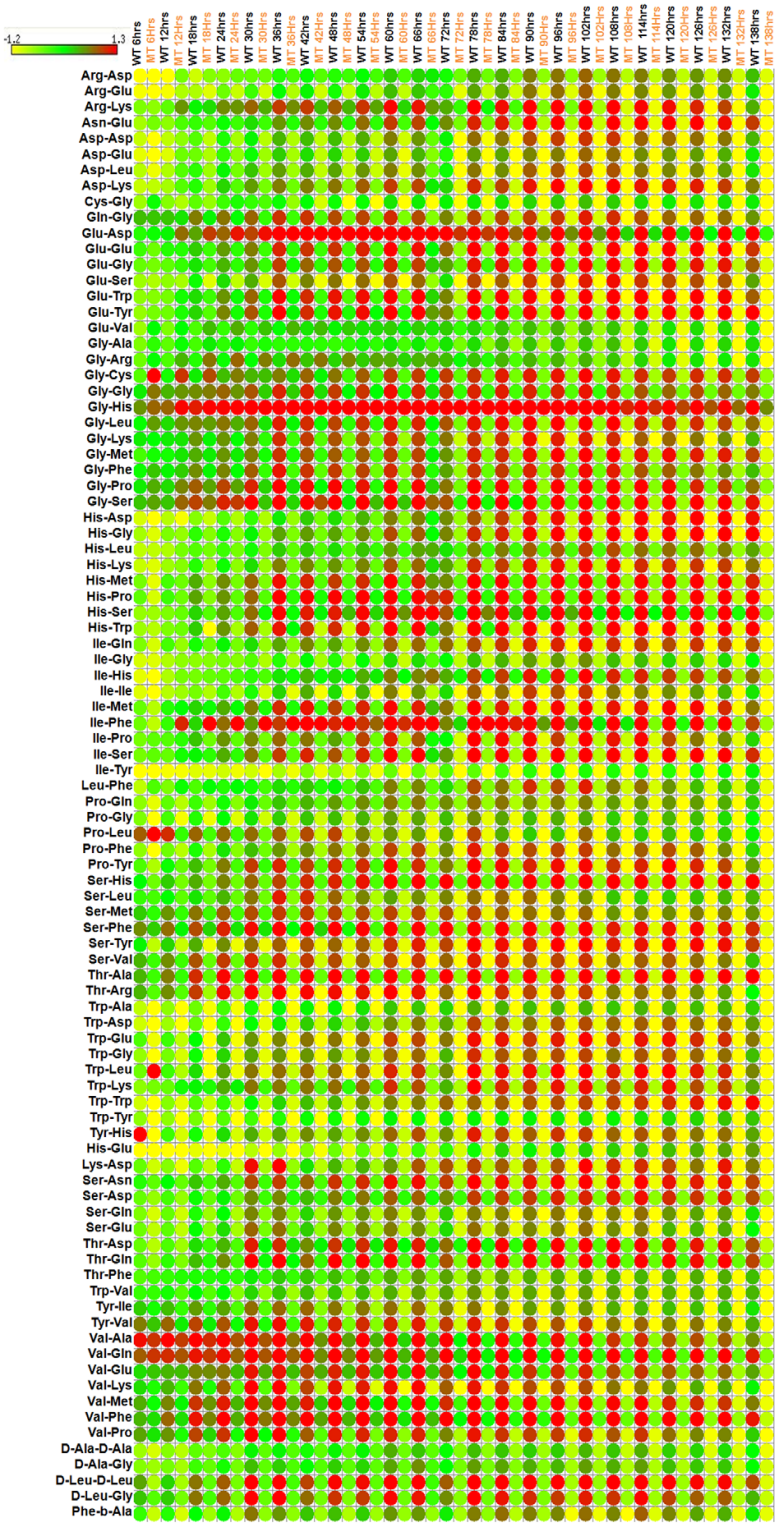
Phenotype array results for peptide nitrogen sources. Normalized values for positive wells in PMs 6–8 are presented. Time course measurement data up to 138 hours is shown. Each row represents a compound and each column represents MAP-WT or *leuD* mutant at a particular time point. Expression range is indicated at top left side of the figure.

Biolog phenotype microarray (PM) for microbial cells is a high-throughput assay for testing utilization of various nutrients. The assay chemistry uses a redox dye to measure respiration of cells [20]. When the bacterial cell is able to utilize a specific test nutrient in a given PM well, redox dye is reduced producing purple color. Since the dye reduction is mostly irreversible under physiological conditions, the color accumulates in the well over a period of hours, amplifying the signal and integrating the amount of respiration over time [20]. There are eight PM plates which test for the utilization of the following nutrient sources; PM1, 2- carbon sources, PM3- nitrogen sources, PM4- phosphorus and sulphur sources, PM5- nutrient supplements, PM6, 7 and 8- peptide nitrogen sources. In this study, we have utilized PMs 1–8 to establish the complete metabolic profile of MAP WT and MAPΔ*leuD*. Of the total of 760 phenotypes tested, 182 tests were positive for MAP WT (Table S3).PM results show that the most preferred carbon source for MAP was Tween 80. Most preferred simple sugars were D-ribose, D-tagatose, D-fucose, D-trehalose and α-D-glucose respectively. Tween 40 and Tween 20 were utilized at the rate similar to D-ribose. Among amino acids, L-leucine was utilized most efficiently as carbon source. The most used nitrogen sources were alloxan and D-mannosamine. The overall results for positive phenotypes in PM plates 1–5 are given in Fig. 2. As shown in Fig. 2, when compared to MAP WT, the ability to utilize most of the nutrients was impaired in MAPΔ*leuD*. A similar trend was observed for peptide nitrogen sources (Fig. 3). Therefore, the deletion of *leuD* gene resulted in the impairment of overall nutrient utilization capacity of MAP. However, one exception was the ability of MAPΔ*leuD* to utilize phosphonoacetic acid while MAP WT did not use this chemical effectively.

### leuD deletion has a large impact on MAP transcriptional response

**Figure 4:**
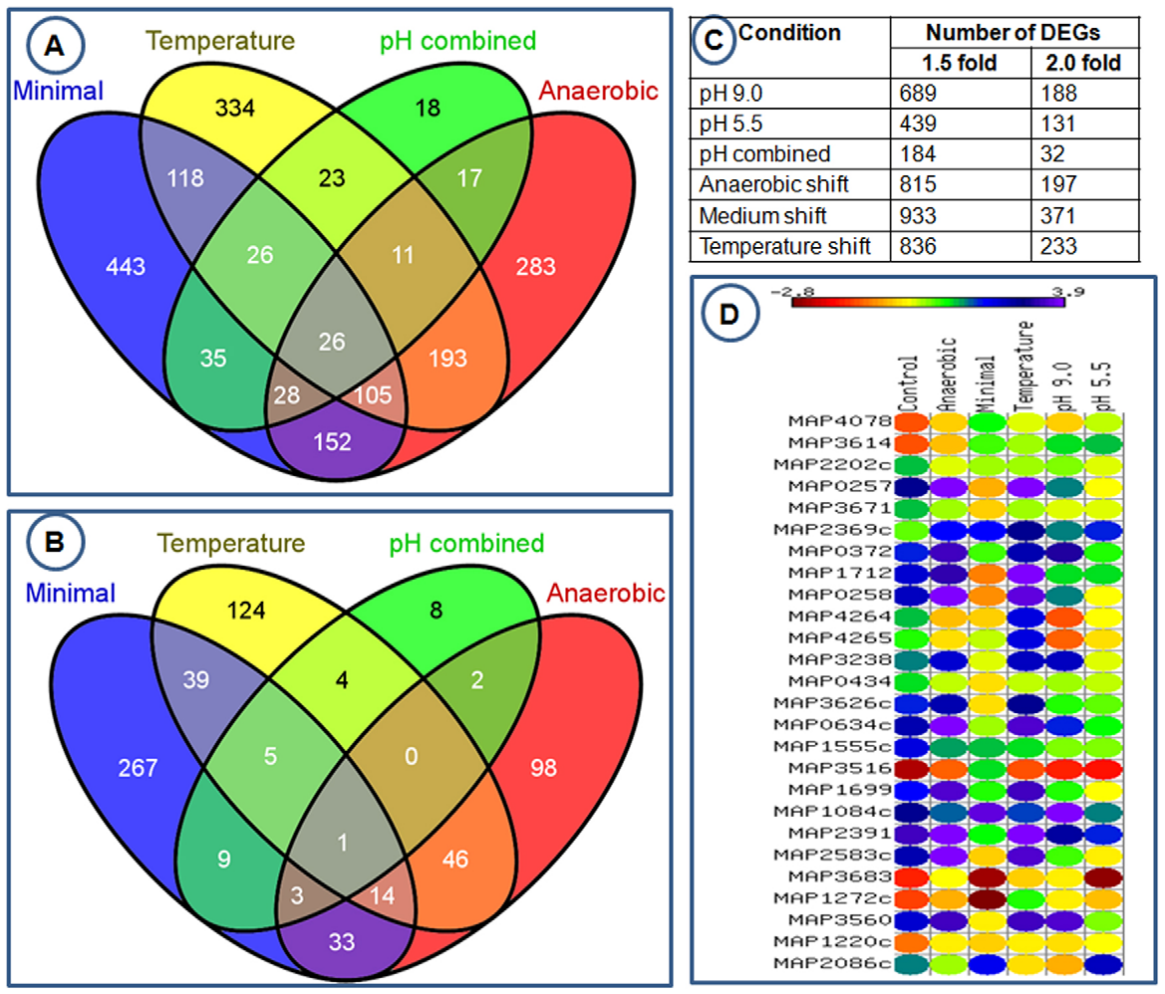
Comparison of Differentially Expressed Genes (DEGs) under various stress conditions. 1A. Comparison of DEGs that change 1.5 fold or more at 5% confidence level. 1B. Comparison of DEGs that change 2.0 fold or more at 5% confidence level. 1C. Comparison of total number of genes at 1.5 fold and 2.0 fold cutoff levels and 1D. Expression pattern of 26 genes that were found to be differentially expressed in all stress conditions at 1.5 fold change cutoff. Each column represents a stress condition and each row represents a gene.

In order to understand the role of *leuD* in MAP stress response, using microarrays we compared the transcriptional profile of mutant and wildtype after subjecting these to different stressors such as shift in pH, nutrient, temperature and anaerobic growth. Similar conditions have been used previously to define the “stressome” of MAP [29]. Anaerobic growth and pH shift were used to mimic the exposure of MAP inside macrophages and in the host abomasum. Growth in the absence of casitone (minimal medium) was used to define the impact on metabolic pathways and temperature shift was used to define general stress response. Two days of growth in the absence of casitone was selected based on the growth pattern of mutant as shown in Fig. 1A. We used three hours of exposure for all other stress conditions as it has been demonstrated to be adequate in a previous study [29]. After transcriptional profiling, two levels of cutoff for defining differentially expressed genes (DEGs) at 5% confidence level were considered. The first set was DEGs with at least 1.5 fold change when compared to control (Fig. 4A) and second set was DEGs with at least 2.0 fold change (Fig. 4B). At both cutoff levels, a large number of DEGs were present in all stress conditions. There were 26 DEGs present in all conditions tested at 1.5 fold cutoff. However, this number diminished to one when the cutoff was raised to two fold levels (Fig. 4B). A comparison of the total number of DEGs at these two cutoff levels is given in Fig. 4C. An overview of expression pattern of 26 DEGs that were found changing in all stress conditions at 1.5 fold cutoff is given in Fig. 4D. The details about the DEGs at both of these cutoff levels are given in Table S4 and S5.

### Deletion of leuD affects transcriptional response of known virulence genes in MAP

**Figure 5:**
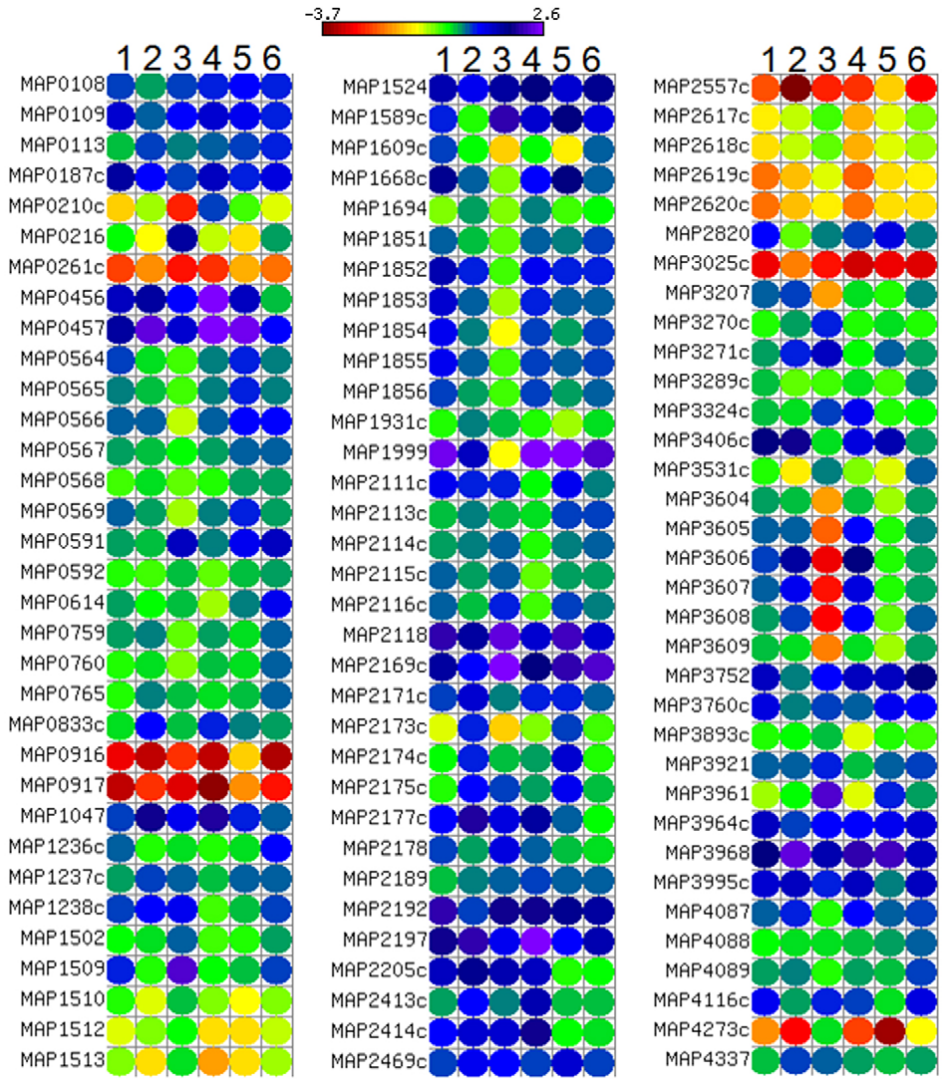
Expression profile of virulence associated MAP genes under various stress conditions. Virulence associated MAP gene definitions were obtained by phylogenomics comparison with other mycobacteria in VFDB. MAP transcriptional expression profiles were then combined with each genes definition. Each column represents a stress condition and each row represents a gene. The stress conditions given as numbers at the top of columns are as follows; 1-Control, 2-Anaerobic, 3-Minimal medium, 4- Temperature, 5-pH 9.0 and 6-pH 5.5.

Comparative phylogenomics is a very useful method for deducing the functions of less studied bacterial genomes by extrapolating data from related species. Although there are not many functional genomics studies in MAP, many related species such as *M. tuberculosis, M. smegamatis, M. bovis, M. avium, and M. leprae* have been studied in more detail. We used the curated phylogenomics data on other mycobacteria from Virulence Factor Database (VFDB) [28] to identify virulence associated genes in MAP(Table S6). We then combined this virulence gene list with microarray expression data from different stress conditions (Fig. 5). As Fig. 5 shows, *leuD* deletion influenced the expression profile of most of the virulence associated genes. It is interesting to note that *leuD* gene itself is annotated as a virulence associated gene in VFDB. Some of the virulence genes in VFDB have also been studied extensively in MAP. For example, the functions of MAP_0216 (*fbpA*), MAP_0187c (*sodA*) and MAP_3921(*sodC*) which were found to be modulated in this study are well defined.

### DEGs are dispersed throughout the MAP gene network

**Figure 6:**
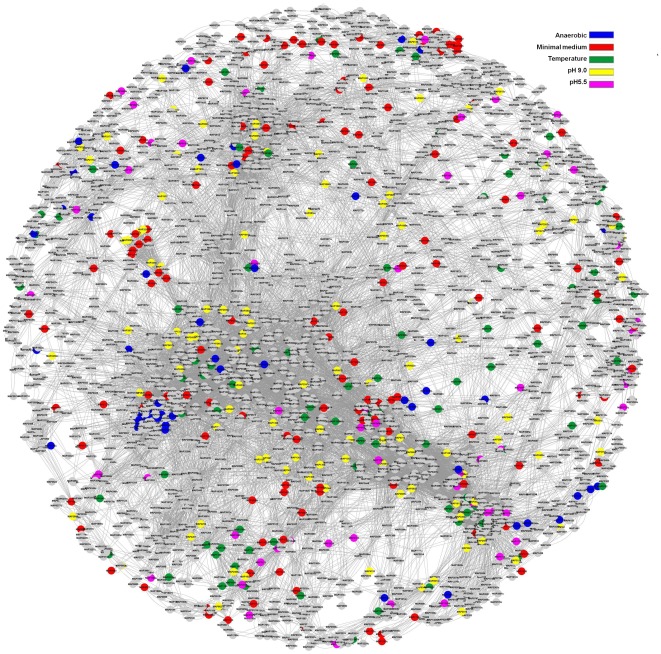
Visualization of Differentially Expressed Genes (DEGs) on MAP gene interaction network. Interaction information of MAP genes was retrieved from STRING database. Interactions with confidence score more than 700 (medium and high confidence interactions) were considered. DEGs with 2.0 fold or more change were then visualized on this network using Cytoscape. DEGs that were changed at different stress conditions are indicated using different color code given at top right side of the figure. Nodes (genes) are indicated by locus tags. Nodes in grey color were not differentially expressed.

Since the Biolog phenotype microarrays and functional microarray data revealed a global effect on MAPΔ*leuD*, we used genome wide systems approach to analyze the DEGs. The interaction data for MAP was derived from STRING [22]. This database contains known and predicted protein-protein interactions which include physical or functional associations [22]. Evidence for these is derived from genomic context, high-throughput experiments, coexpression and literature mining [22]. After combining different types of evidences for each interaction, only interactions with score of 700 or more was considered. This score cut off represented medium and high confidence interactions for MAP. The final interaction network contained 3,350 nodes (genes) and 28,932 edges (interactions). When 2.0 fold DEGs were visualized on this network, we find that they are scattered throughout the interaction network (Fig. 6). If 1.5 fold DEGs are considered, this effect is more pervasive (data not shown). This reveals that leucine metabolism is tightly integrated with the whole cellular networks and would explain the severe impairment of MAPΔ*leuD's* ability to utilize most of the nutritional sources in Biolog phenotype arrays.

**Figure 7:**
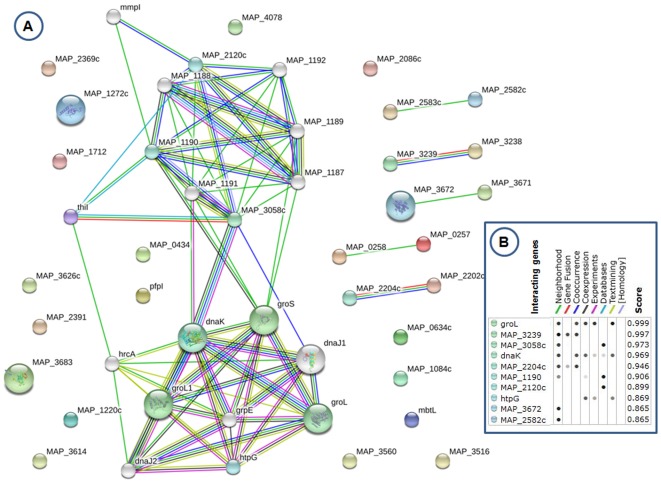
Gene interaction network of 26 genes that were found to be changing at all stress conditions. 7A. STRING database was searched using list of 26 genes that were found to be differentially expressed 1.5 fold or more at all stress conditions tested. Nodes (genes) are indicated by locus tag or gene name. Edges (interactions) are denoted by the color code for source of evidence and are indicated at the top side of Fig. 7B in the inset. 7B. List of predicted interaction partners for these 26 DEGs. List of predicted interaction partners are given as locus tag or gene name in the left most column. The evidence source for each interaction is represented as columns. Cumulative evidence score is given in the right most columns.

We further attempted to elucidate the role of 26 DEGs that were found changing at least 1.5 fold in all stress conditions. We used manual search of STRING database to find the interaction partners for these genes (Fig. 7A). This analysis predicted the following interaction partners with very high confidence evidence scores; *groL*, MAP_3239, MAP_3058c, *dnaK* and MAP_2204c (Fig. 7B). Cumulative results of this analysis suggest that 26 DEGs found to be changing in all stress conditions are involved in heat shock response and maintenance of cell viability.

### leuD deletion affects several MAP cellular pathways, particularly the fatty acid biosynthesis pathways

**Figure 8:**
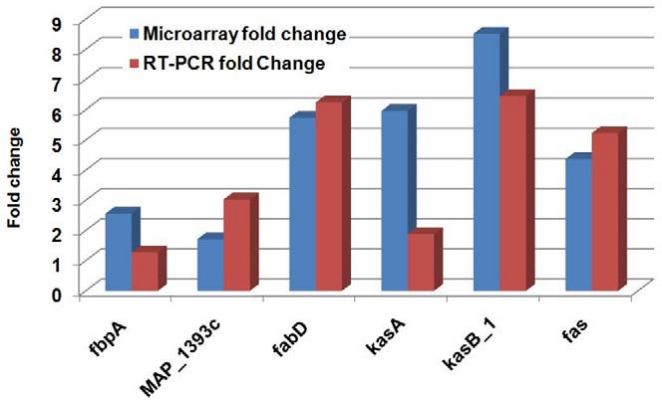
Comparison of gene expression fold change in key genes associated with mycolate biosynthesis pathway. Genes associated with mycolate biosynthesis were identified using pathway annotation in Biocyc and by ortholog mapping of genes as defined in mycolate biosynthesis pathway of *M. tuberculosis*. Gene expression fold change was measured using both microarrays, and qRT-PCR.

Since *leuD* gene is part of super pathway of leucine, valine and isoleucine biosynthesis, it is natural to reason that deletion of this gene will have major impact on other cellular pathways of MAP. For analyzing the DEGs in the context of cellular pathways, we used the Biocyc [30] pathway reconstruction of MAP. Pathway tools software [25] was used to visualize 2.0 fold changing DEGs on the cellular pathways (Fig. S1). As expected, fatty acid biosynthesis II, palmitate biosynthesis II, mycolate biosynthesis, fatty acid beta-oxidation, acetate degradation and oleate beta-oxidation pathways were found to be differentially expressed. Other differentially expressed pathways included pathway of assimilatory sulfate reduction, malonate degradation, acetyl-CoA fermentation to butyrate I, glutaryl-CoA degration, arginine biosynthesis II, and pantothenate and coenzyme A biosynthesis I. We used the pathway enrichment tool in Pathway tools to identify statistically significant pathways changes associated with each stress condition. The result of this analysis is given Table 1. As evident in Table 1, there were a total of 53 pathways that were associated with different stress conditions. The following main compounds are either produced or consumed by leucine biosynthesis pathway; (2R, 3S)-3-isopropylmaleate, (2S)-2-isopropyl-3-oxosuccinate, (2S)-2-isopropylmaleate, 2-oxoisovalearate, 4-methyl-2-oxopentanoate, and L-leucine. In addition, the following side compounds in leucine biosynthesis pathway forms links with other pathways; 2-oxoglutarate, acetyl-CoA, coenzyme A, NAD+, NADH and L-glutamate. As shown in Fig.S1, the main effect of *leuD* deletion is on the pathways dependent on or linked to altered levels of needed primary or side compounds produced or consumed by the leucine biosynthetic pathway. For example, we found that key mycolate biosynthetic pathway genes are modulated in different stress conditions, particularly during growth in minimal medium. Our results measuring mRNA levels mirrored changes in gene activity observed in microarray tests by performing qRT-PCR on the same set of genes in this particular pathway (Fig. 8) showing that both microarray and qRT-PCR test results are in agreement with each other.

**Table 1:** List of differentially enriched MAP pathways under different stress conditions.

Pathway Name	P value	Associated Genes	Stress Condition
NAD biosynthesis I (from aspartate)	0.002059698	nadA, nadB	Anaerobic
NAD Biosynthesis	0.003066353	nadA, nadB	Anaerobic
NAD Metabolism	0.004260643	nadA, nadB	Anaerobic
Aromatic Compounds Degradation	0.015659954	catB	Anaerobic
Catechol Degradation	0.015659954	catB	Anaerobic
catechol degradation to beta;-ketoadipate	0.015659954	catB	Anaerobic
Aerobic Respiration	3.06E-13	nuoA, nuoB, nuoC, nuoD, nuoE, nuoG, nuoH, nuoI_2, nuoJ, nuoM, nuoN, sdhA	Minimal Medium
aerobic respiration (cytochrome c)	3.06E-13	nuoA, nuoB, nuoC, nuoD, nuoE, nuoG, nuoH, nuoI_2, nuoJ, nuoM, nuoN, sdhA	Minimal Medium
Respiration	7.27E-09	nuoA, nuoB, nuoC, nuoD, nuoE, nuoG, nuoH, nuoI_2, nuoJ, nuoM, nuoN, sdhA	Minimal Medium
Generation of Precursor Metabolites and Energy	3.53E-05	aceA, fadA_2, nuoA, nuoB, nuoC, nuoD, nuoE, nuoG, nuoH, nuoI_2, nuoJ, nuoM, nuoN, sdhA	Minimal Medium
fatty acid biosynthesis initiation II	0.001048242	kasA, kasB_1, kasB_2	Minimal Medium
fatty acid biosynthesis initiation III	0.001048242	kasA, kasB_1, kasB_2	Minimal Medium
cis-vaccenate biosynthesis	0.002628078	kasA, kasB_1, kasB_2, MAP2861	Minimal Medium
fatty acid elongation – saturated	0.003936254	kasA, kasB_1, kasB_2, MAP2861	Minimal Medium
superpathway of unsaturated fatty acids biosynthesis (*E. coli*)	0.003936254	kasA, kasB_1, kasB_2, MAP2861	Minimal Medium
histidine biosynthesis	0.01744924	hisA, hisF, hisH	Minimal Medium
Histidine Biosynthesis	0.01744924	hisA, hisF, hisH	Minimal Medium
Fatty Acid Biosynthesis	0.0366217	fadD2, fadD5, kasA, kasB_1, kasB_2, MAP2861	Minimal Medium
Sulfate Reduction	0.003066353	cysH_1, nirA_1	pH 5.5
sulfate reduction I (assimilatory)	0.003066353	cysH_1, nirA_1	pH 5.5
Sulfur Compounds Metabolism	0.004260643	cysH_1, nirA_1	pH 5.5
Inorganic Nutrients Metabolism	0.005638148	cysH_1, nirA_1	pH 5.5
acetoacetate degradation (to acetyl CoA)	0.010826666	fadA2, fadA_2	pH 5.5
Degradation/Utilization/Assimilation	0.014504217	catB, cysH_1, fadA2, fadA_2, nirA_1	pH 5.5
Aromatic Compounds Degradation	0.015659954	catB	pH 5.5
Catechol Degradation	0.015659954	catB	pH 5.5
catechol degradation to beta;-ketoadipate	0.015659954	catB	pH 5.5
Acetyl-CoA Fermentation to Butyrate	0.025580868	fadA2, fadA_2	pH 5.5
acetyl-CoA fermentation to butyrate II	0.025580868	fadA2, fadA_2	pH 5.5
Arginine Biosynthesis	1.93E-09	argB, argC, argD, argF, argG, argH, argJ	pH 9.0
arginine biosynthesis II (acetyl cycle)	1.93E-09	argB, argC, argD, argF, argG, argH, argJ	pH 9.0
arginine biosynthesis I	5.92E-08	argB, argC, argD, argF, argG, argH	pH 9.0
Aerobic Respiration	7.31E-05	nuoA, nuoB, nuoC, nuoD, nuoI_2	pH 9.0
aerobic respiration (cytochrome c)	7.31E-05	nuoA, nuoB, nuoC, nuoD, nuoI_2	pH 9.0
ornithine biosynthesis	1.48E-04	argB, argC, argD	pH 9.0
Individual Amino Acids Biosynthesis	5.22E-04	argB, argC, argD, argF, argG, argH, argJ, leuA	pH 9.0
Amino Acids Biosynthesis	0.001009225	argB, argC, argD, argF, argG, argH, argJ, leuA	pH 9.0
Respiration	0.001519393	nuoA, nuoB, nuoC, nuoD, nuoI_2	pH 9.0
Other Amino Acid Biosynthesis	0.002784306	argB, argC, argD	pH 9.0
Generation of Precursor Metabolites and Energy	0.027987104	aceA, nuoA, nuoB, nuoC, nuoD, nuoI_2	pH 9.0
citrulline degradation	0.035794184	argF	pH 9.0
Miscellaneous Amino Acids Degradation	0.035794184	argF	pH 9.0
Coenzyme A Biosynthesis	7.01E-05	MAP0458, panB, panC, panD	Temperature
pantothenate and coenzyme A biosynthesis I	7.01E-05	MAP0458, panB, panC, panD	Temperature
Pantothenate Biosynthesis	2.37E-04	MAP0458, panB, panC	Temperature
phosphopantothenate biosynthesis I	2.37E-04	MAP0458, panB, panC	Temperature
arginine biosynthesis I	0.001847533	argD, argG, argH	Temperature
Arginine Biosynthesis	0.002590142	argD, argG, argH	Temperature
arginine biosynthesis II (acetyl cycle)	0.002590142	argD, argG, argH	Temperature
beta; Alanine Biosynthesis	0.03131991	panD	Temperature
beta;-alanine biosynthesis III	0.03131991	panD	Temperature
Phenylalanine Degradation	0.03131991	phhB	Temperature
phenylalanine degradation I (aerobic)	0.03131991	phhB	Temperature

Differentially expressed genes displaying a 2.0 fold or more increase in microarray measurement levels were imported into Pathway tools software. From this gene list, pathways that were differentially enriched under different stress conditions were identified using differential enrichment tool as implemented in Pathway tools. A p-value of 0.05 was used as a cutoff.

### MAPΔleuD has reduced cellular lipid profile

Since the transcriptional analysis suggested that the fatty acid synthesis pathways in MAPΔleuD are affected, we verified this possibility using FAME analysis of MAP-WT and MAPΔ*leuD*. Sherlock® FAME analysis system uses gas chromatography and sophisticated pattern recognition software to identify over 300 fatty acids in the range of 9 to 20 carbons in length. The total amount of fatty acids detected by FAME analysis of MAP-WT and MAPΔ*leuD* were 396831 and 275561 units, respectively. This corresponds to 30% reduction of fatty acid content in mutant cells in equivalent amount of biomass. The most abundant fatty acids were 18:1 w9c, 16:00 and 16:1 w9c (Table 2). Some peaks that were present in MAP-WT were missing in MAPΔ*leuD*. The FAME spectrum analysis result for MAP-WT and MAPΔ*leuD* is given in Fig. S2. Overall, the FAME analysis results are in agreement with computational analysis of transcriptional data suggesting that deletion of *leuD* leads to alteration of cellular lipid profile of MAP.

**Table 2:** Result of FAME analysis of MAP-WT and MAPΔ*leuD*.

Fatty Acid	MAP WT			MAPΔ*leuD*		
	Response	% of Total	ECL Deviation	Response	% of Total	ECL Deviation
9:00	380	0.12	−0.001	ND	ND	ND
12:00	1086	0.29	0.001	698	0.27	−0.002
14:00	13295	3.31	0.000	6540	2.34	0.000
15:0 anteiso	498	0.12	0.001	433	0.15	0.002
15:1 w8c	498	0.12	0.006	ND	ND	ND
15:00	2272	0.55	0.002	1135	0.4	0.003
16:0 iso	512	0.12	0.004	375	0.13	0.003
16:1 w9c	29418	7.02	−0.001	28400	9.76	0.000
Summed Feature 3	14123	3.37	0.003	11149	3.83	0.007
16:1 w5c	684	0.16	0.001	596	0.2	0.006
16:00	82726	19.68	0.002	55175	18.9	0.002
Summed Feature 4	719	0.17	0.000	ND	ND	ND
17:0 iso	877	0.21	−0.012	979	0.33	−0.015
17:0 anteiso	1983	0.47	0.000	1367	0.46	0.001
17:1 w7c	12544	2.95	0.001	5088	1.72	0.003
17:00	1845	0.43	0.002	796	0.27	0.001
18:1 w9c	147798	34.35	0.001	104829	35.09	0.002
18:00	11564	2.68	−0.002	7101	2.37	0.000
10-methyl 18:0 TBSA	20954	4.85	0.001	17755	5.91	0.001
19:0 iso	1735	0.4	0.004	2293	0.76	0.003
Summed Feature 8	75147	17.32	−0.003	46049	15.28	−0.004
20:1 w9c	1837	0.42	0.008	1354	0.45	0.005
20:1 w7c	1314	0.3	0.001	939	0.31	−0.002
20:00	2561	0.59	0.000	2069	0.68	0.000
**Total Fatty acid amount**	**396831**			**275561**		

Both mutant and wildtype strains were cultured in 7H9 medium supplemented with casitone until mid-log phase. Mutant was then shifted 7H9 medium without casitone for 12 hours. Frozen cell pellet was then tested for fatty acids using FAME analysis. Peaks in the GC spectrum were identified using Sherlock® V 6.1.

## Discussion

### leuD is intricately linked to global MAP metabolism

In this study, for the first time, we report the complete nutrient utilization profile of MAP using phenotype arrays. One of the surprising results we obtained is that Tween 80 was used by MAP as carbon source and was even more preferred than most common simple sugars such as D-ribose and α-D-glucose (Table S3). Other Tween types such as Tween 40 and 20 also were utilized well as carbon sources. Generally mycobacterial culture media are supplemented with small amounts of Tween as a surfactant to avoid cell clumping and more quantities are believed to be toxic. Addition of Tween 80 in the medium has been reported to change the susceptibility of *M. avium- M. intracellulare* complex [31]. On the other hand, Tween 80 has been reported to be hydrolyzed *by M. bovis* [32] and *M. avium* subsp. *paratuberculosis* [33]. Further, *M. smegmatis* grown in the presence of Tween 80 synthesizes series-2 type glycolipids [34]. It has also been reported that bacteria such as *Microthrix parvicella* uses the oleic acid moiety of Tween 80 as carbon and energy source [35]. Therefore, it is reasonable to assume that MAP also uses oleic acid component of Tween 80 in the same manner. PM results showed that while L-leucine is used as carbon source by MAP-WT, the MAPΔ*leuD* lost this ability. This indicates that external supplementation of leucine is not as efficient as native biosynthesis of this amino acid and could explain why supplementation of casitone did not restore growth rates of MAPΔ*leuD* to those of wildtype MAP. Among pentose sugars such as D-trehalose that was shown to be used as carbon source have well recognized role in mycobacteria. For example, trehalose is an essential component of mycolic acid [26]. However, we could not find annotated enzymes for the metabolism of sugars such as L-lyxose, D-tagatose, and D-fucose. Rather than the absence of transferases and isomerases required for the utilization of these sugars, it most likely indicates the need for annotating these genes correctly in MAP genome. Reason for this suggestion is that these enzymes have been identified in related mycobacteria such as *M. smegmatis* [36] and *M. avium* [37]. Since there is very limited literature regarding nutrient sources such as alloxan, xanthine and peptide nitrogen sources, we could not link the metabolism of these compounds and the enzymes and pathways in MAP. The overall observation that MAPΔ*leuD* lost the ability to use most of the nutritional sources reveals the intricate connection between MAP metabolism and virulence. The only instance of reversal of nutrient utilization was the ability of MAPΔ*leuD* to utilize phosphonoacetic acid. Although it has been reported that some mycobacteria utilizes phosphonoacetate as sole carbon, phosphorus and energy source [38], further studies are required to understand the importance of this ability reversal. In addition to revealing the intricate link between *leuD* and global metabolism of MAP, the PM data we report here can have several other uses, including (i) improvement of bacterial metabolic and systems models [39], [40], [41], [42], (ii) assessing the accuracy of genome annotation [43], and (iii) developing new improved culture media for bacteria [44].

### Effect of leuD deletion on MAP transcriptional response to stress

In addition to studying the effect of *leuD* deletion at the phenotype level, we also compared the transcriptional response of MAP-WT and MAPΔ*leuD*. In the past there have been a very limited number of studies examining the MAP transcriptional responses to deletion mutants [29], [45], [46], [47], [48]. However, this is the first report where MAP transcriptional response is analyzed after deletion of a metabolically important MAP gene. As expected, we found that a large number of genes were modulated under different stress conditions. The 26 genes that were found to be modulated in all stress conditions in our study can be candidates of further characterization. Although the fold change cutoff for these genes is 1.5 fold, the fact that these genes are differentially expressed in all conditions indicates their importance in MAP environmental responses. The majority of these was hypothetical proteins and could be candidates for further characterization. While *groES* (MAP_4264) and *groEL* (MAP_4265) were up-regulated during temperature shift, they were found to be down-regulated several fold in all other conditions. The heat shock proteins were also found to be among the DEGs in the previous MAP “stressome” study [29]. Two other annotated DE genes common to all conditions were *pfpl* (MAP_0372) and *thil* (MAP_1699). It has been reported that *pfpl* plays an anti-mutator role and provides general stress protection *Pseudomonas aeruginosa* [49]. In *Salmonella enterica* serovar Typhimurium it has been shown that ThiL has dual role in *de novo* biosynthesis and in salvage of exogenous thiamin [50]. We used an integrated approach of using systems level gene interaction networks and phylogenomics to understand the link between *leuD* deletion and metabolic and transcriptional responses. As Fig. 6 shows, the DEGs were distributed throughout the MAP gene network. This would explain why *leuD* deletion had such huge effect on the global phenotype. The dispersed nature of DEGs in the MAP gene network also explains why a large number of virulence associated genes were also modulated in the stress conditions tested.

### Effect of leuD deletion on MAP pathways

Since *leuD* deletion leads to the abolition of leucine biosynthesis, it is not surprising to see that a total of 53 pathways significantly modulated in the stress conditions studied. Previously using a mouse model, we have demonstrated that vaccination of MAPΔ*leuD* strain provided significant protective efficacy against a MAP virulent strain challenge [19]. The pathway analysis results suggested that this protective efficacy could be due the effect of *leuD* deletion on metabolic pathways associated with fatty acids, particularly mycolate biosynthesis (Fig. S1). Our results suggest that in MAPΔ*leuD*, reduced activity of leucine biosynthesis pathway leads to diminished levels of 2-oxoglutarate, acetyl-CoA, coenzyme A, NAD+, and NADH. These compounds are required to complete several steps in synthesis of mycolic acid and other fatty acids. We verified this possibility experimentally by estimating the levels of fatty acids in MAP WT and MAPΔ*leuD*. Although the overall spectrum (Fig. S2) and fatty acid peaks appear similar in both strains, FAME analysis results shows that in MAPΔ*leuD* there is about 30% reduction in fatty acid levels (Table 2). The reason for similarity in peaks in the FAME spectrum is that initially MAP-WT and MAPΔ*leuD* were grown to mid-exponential-phase in 7H9 medium and MAPΔ*leuD* was switched to minimal medium. This initial culturing of both strains in the same medium seems to be resulting in similar spectrum appearance. However, the 30% reduction in overall fatty acid levels does support our pathway analysis results that *leuD* deletion has a major effect on pathways associated with fatty acids. It is well known that the membrane lipid profile is important in mycobacterial survival and virulence [26], [51], [52], [53]. In addition to this, *leuD* deletion also appears to be influencing mycolate biosynthesis pathway be modulating genes such as *fbpA*. Mycolic acids are synthesized as two alkyl chains by enzymes similar to those that assemble fatty acids and these chains are fused and modified numerous times to generate a complex family of lipids that are incorporated into the cell envelope [54], [55], [56]. Secreted proteins FbpA, FbpB, and FbpC attach mycolic acids to arabinogalactan, generating mycolic acid methyl esters (MAME) or trehalose, generating α,α′-trehalose dimycolate (TDM) and is essential for mycobacterial virulence [56]. Therefore, the mechanism behind the attenuation of MAPΔ*leuD* strain could be due to reduced levels of cellular fatty acids and mycolic acids. Since mycolic acid biosynthesis pathway is generally conserved in Mycobacteria, it is natural to assume that the link between leuD metabolism and mycolic acid biosynthesis we indentified in MAP could be also be present in other Mycobacteria such as *M. tuberculosis*. Further, several of the MAP phenotypes we defined here, could also be conserved in other Mycobacteria.

## Supporting Information

Table S1 Details about primers used for qRT-PCR.
(XLSX)

Table S2 Ortholog mapping of mycolate biosynthesis genes between *M. tuberculosis* (MTB) and MAP.
(XLSX)

Table S3 Complete results of PM tests that are positive for PM panels 1–8.
(XLSX)

Table S4 list of microarray 1.5 fold changing genes.
(XLSX)

Table S5 List of microarray 2.0 fold changing genes.
(XLSX)

Table S6 List of virulence associated genes in MAP.
(XLS)

Figure S1 Visualization of 2.0 fold changing DEGs on MAP pathways. The DEGs are highlighted on pathways according to the following color codes; (i) Blue-pathways not associated with DEGs, (ii) red- pathways mapped to pH 5.5 associated DEGs, (iii) orange- pathways mapped to pH 9.0 associated DEGs, (iv) green- pathways mapped to temperature associated DEGs, (v) pink- pathways mapped to anaerobic associated DEGs, (vi) brown- pathways mapped to minimal medium associated DEGs. Pink lines indicate pathways consuming metabolites from leucine pathway.
(TIF)

Figure S2 FAME spectrum of MAP-WT and MAPΔ*leuD*. Raw gas chromatography spectra for both mutant and wildtype strains are shown. Peaks in the GC spectrum were identified using Sherlock® V 6.1.
(TIF)
