# Supplementary figures and images for: Phenotypic and Transcriptomic Response of Auxotrophic Mycobacterium avium Subsp. paratuberculosis leuD Mutant under Environmental Stress
Source: PLoS One. 2012 Jun 4;7(6):e37884. doi: 10.1371/journal.pone.0037884 (PMC3366959; doi:10.1371/journal.pone.0037884)

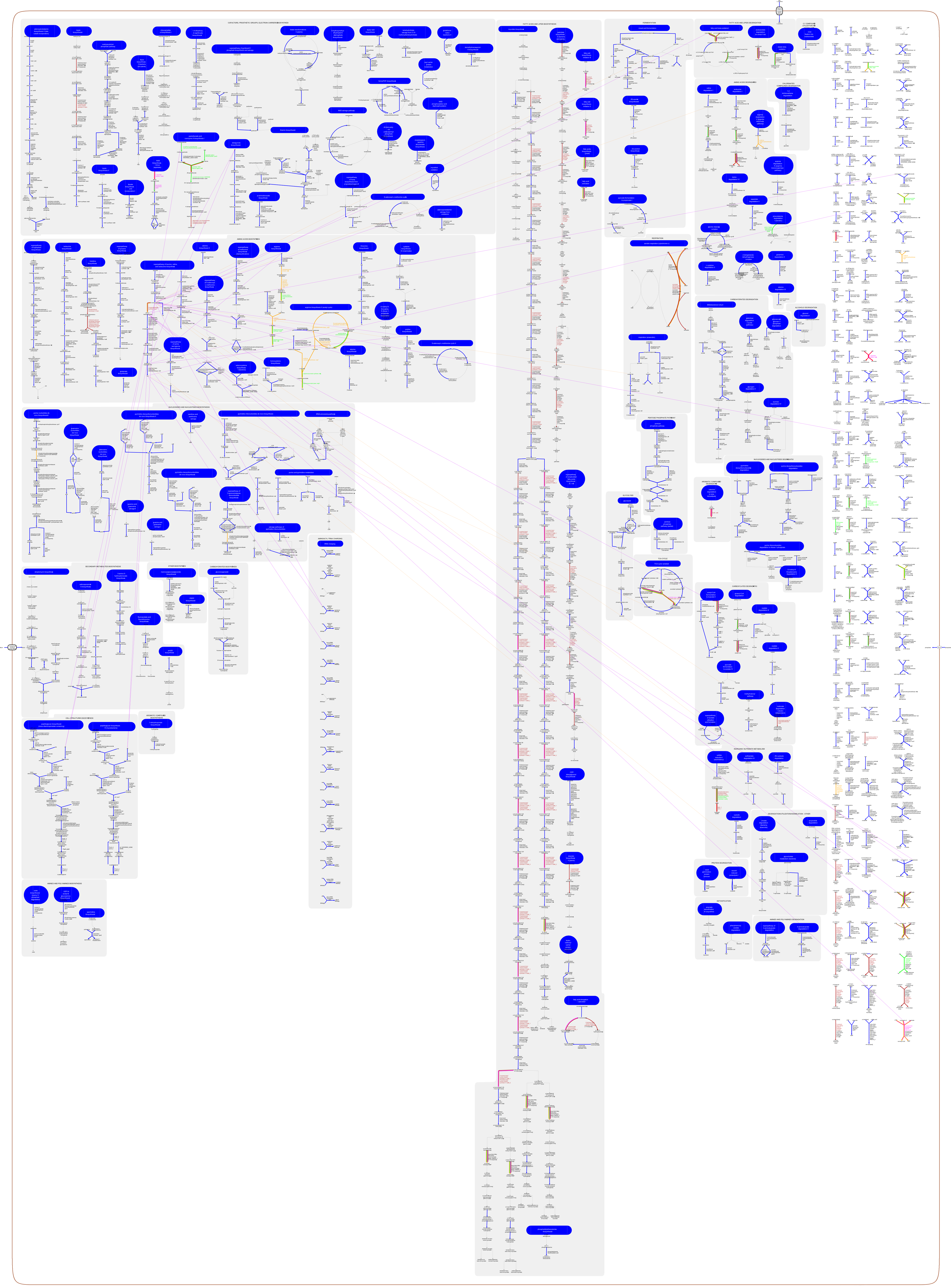

Supplement: Figure S1 — Visualization of 2.0 fold changing DEGs on MAP pathways. The DEGs are highlighted on pathways according to the following color codes; (i) Blue-pathways not associated with DEGs, (ii) red- pathways mapped to pH 5.5 associated DEGs, (iii) orange- pathways mapped to pH 9.0 associated DEGs, (iv) green- pathways mapped to temperature associated DEGs, (v) pink- pathways mapped to anaerobic associated DEGs, (vi) brown- pathways mapped to minimal medium associated DEGs. Pink lines indicate pathways consuming metabolites from leucine pathway. (TIF) [file pone.0037884.s007.tif]

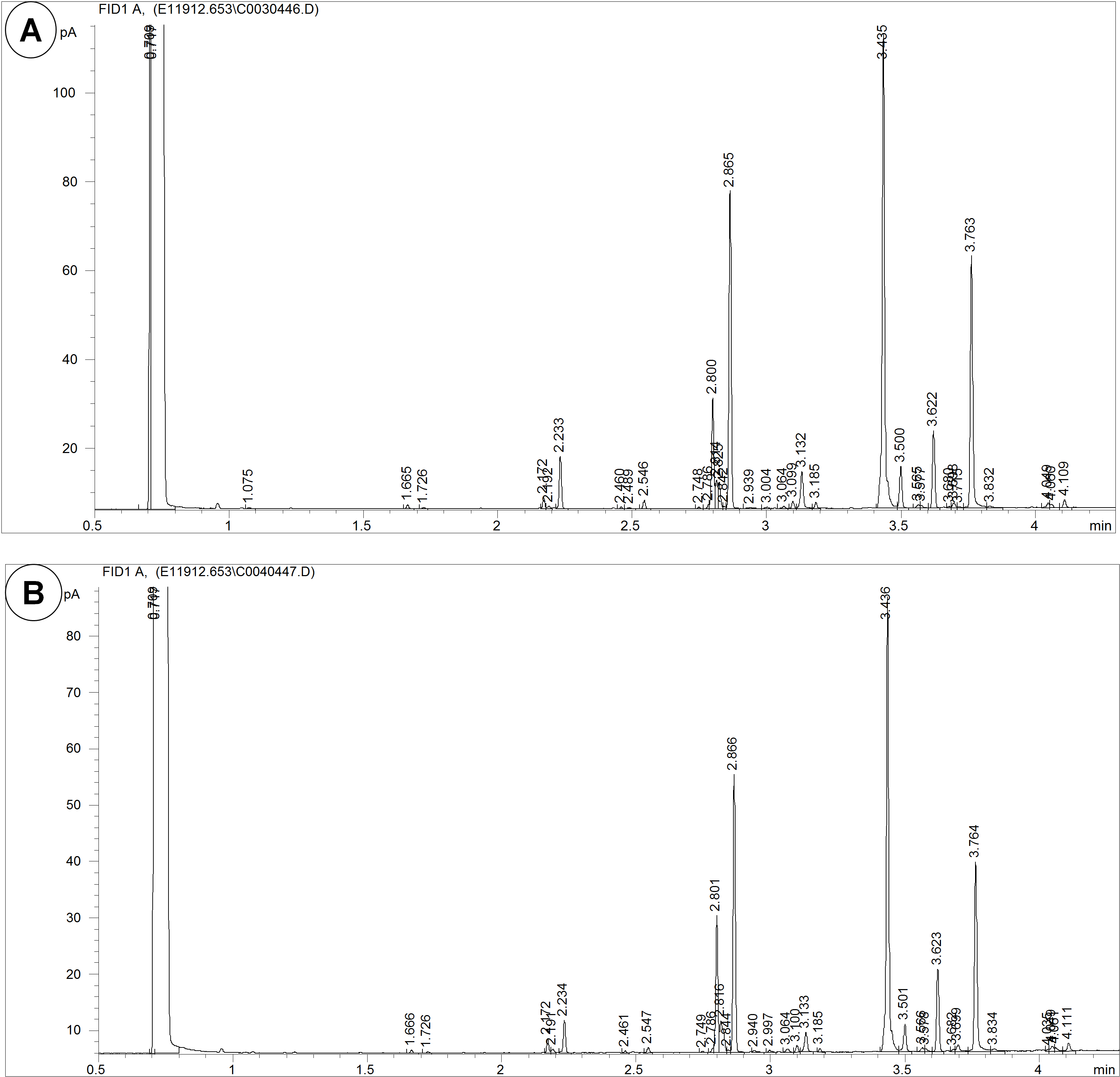

Supplement: Figure S2 — FAME spectrum of MAP-WT and MAPΔleuD. Raw gas chromatography spectra for both mutant and wildtype strains are shown. Peaks in the GC spectrum were identified using Sherlock® V 6.1. (TIF) [file pone.0037884.s008.tif]
